# Special Issue: “New Diagnostic and Therapeutic Tools against Multidrug-Resistant Tumors (STRATAGEM Special Issue, EU-COST CA17104)”

**DOI:** 10.3390/cancers14225491

**Published:** 2022-11-08

**Authors:** M. Helena Vasconcelos, Catherine Passirani, Chiara Riganti

**Affiliations:** 1 i3S—Instituto de Investigação e Inovação em Saúde, Universidade do Porto, Rua Alfredo Allen 208, 4200-135 Porto, Portugal; 2 IPATIMUP—Institute of Molecular Pathology and Immunology, University of Porto, Rua Júlio Amaral de Carvalho 45, 4200-135 Porto, Portugal; 3 Department of Biological Sciences, FFUP—Faculty of Pharmacy, University of Porto, Rua de Jorge Viterbo Ferreira 228, 4050-313 Porto, Portugal; 4 Micro et Nanomédecines Translationnelles, MINT, UNIV Angers, UMR INSERM 1066, UMR CNRS 6021, SFR ICAT, 49000 Angers, France; 5 Department of Oncology, University of Torino, Via Santena 5/bis, 10126 Torino, Italy; 6 Interdepartmental Research Center for Molecular Biotechnology “G. Tarone”, University of Torino, Via Nizza 52, 10126 Torino, Italy

Cancer drug resistance, either intrinsic or acquired, often causes treatment failure and increased mortality [1]. Multidrug resistance (MDR) is characterized by cross-resistance to several anticancer drugs with distinct structures and mechanisms of action [2,3]. Multi-disciplinary approaches are necessary to better understand MDR’s underlying mechanisms [4,5,6,7] and identify predictive biomarkers [8], new therapeutic targets [9] and new drugs and formulations [10,11].

The European COST (Cooperation in Science and Technology) Action STRATAGEM “New Diagnostic and Therapeutic Tools against Multidrug-Resistant Tumors” was initiated in April 2018, as a multidisciplinary open consortium studying the diagnostic, therapeutic and toxicological challenges associated with MDR tumors. Indeed, multiple tools must be used to overcome MDR, including molecular modelling [12], high-throughput bioinformatic analyses [9,13], biochemical and pharmacological assays together with advanced technological tools [14], rational design and synthesis of new synthetic or natural bioactive compounds [10,15,16] and preparation of formulations using nanocarriers to improve drug solubilization, selectivity and anti-cancer action [11].

This Special Issue of *Cancers* publishes the latest innovative original research and review articles from members of the STRATAGEM COST Action (CA17104) program. The papers include four review articles and five original research articles.

Docetaxel, a microtubule-stabilizing taxane, is used in the treatment of metastatic castration-resistant prostate cancer. However, resistance often occurs, limiting treatment response [17]. Thus, the identification of acquired docetaxel resistance mechanisms in prostate cancer is of utmost relevance. In the study of Lima et al. [18], docetaxel-resistant prostate cancer cell lines were established and characterized, and genome-wide gene expression profiling was performed, resulting in the identification of the presence of multiple mechanisms of drug resistance. The authors suggest that counteracting these mechanisms could provide an approach to re-sensitize docetaxel-resistant prostate cancer.

One major obstacle encountered in the eradication of MDR cells is the hyperactivation of pro-survival pathways dependent on PI3K/Akt and anti-apoptotic proteins, as those of Bcl-2 family (Bcl-2, Bcl-xL, Mcl-1), paralleled by the overexpression of mutated TP53, which loses its function of inducer of pro-apoptotic genes, and of Inhibitors of Apoptosis Proteins (IAPs), as XIAP, c-IAP1 and c-IAP2, BRUCW, NAIP, ILP-2 and MLIAP, as extensively highlighted in the review by Neophytou et al. Notably, the authors underline as the extensive deregulation of anti-apoptotic/pro-apoptotic proteins is not only a tumor-dependent event, but also a tumor-microenvironment (TME)-dependent event. Indeed, cancer-associated fibroblasts (CAFs) secrete interleukins or extracellular vesicles containing soluble factors that activate anti-apoptotic pathways in MDR cancer cells. If the potent arsenal of anti-apoptotic actors makes MDR cells harder to be eradicated by common chemotherapeutic drugs, they also offer new therapeutic opportunities, since in recent years several small molecules or natural products targeting Bcl-2, MDM2 or PI3K/mTOR axis have been successfully developed and proved to restore the cytotoxic effects of chemotherapeutic drugs in different solid cancers [19]. This research opens the way to test these new combinations in clinical settings.

Resistance to small molecules is not limited to solid tumors; indeed, hematological diseases, such as chronic myeloid leukemia (CML), showed a high degree of resistance to some tyrosine kinase inhibitors (TKIs), such as Imatinib, Dasatinib, Nilotinib, Bosutinib and Ponatinib [20]. The work of Alves et al. extensively reviewed the different mechanisms, either dependent or independent on the BCR-ABL1 aberration, determining resistance to TKI in CML. Several combinations based on TKIs and small molecules targeting other pathways critical for CML cell survival are currently in clinical trials. Moreover, the study also highlights the future possibilities of conceiving TKIs and/or combination treatments effective against resistant cells, exploiting the most recent techniques in molecular profiling (such as next-generation sequence and digital droplet PCR) and in artificial intelligence to achieve a high-throughput in silico drug design [21].

It is known that some TKIs increase the sensitivity of MDR cancer cells to chemotherapy, by interacting with ABC transporters, either as their substrates or inhibitors [22]. In a study conducted by Podolski-Renić et al. [23], the potential of novel TKI pyrazolo[3,4-d]pyrimidines and their prodrugs to inhibit P-glycoprotein (P-gp) was investigated. Interestingly, a collateral sensitivity effect was observed in the MDR cell lines. The compounds inhibited the ATPase activity of P-gp, with one of the prodrugs displaying the highest inhibition effect. Importantly, prodrugs sensitized MDR cancer cells to doxorubicin and paclitaxel in a concentration-dependent manner. Thus, this study provides an interesting strategy for reversing P-gp-mediated MDR.

One strategy to overcome resistance to both target therapies and classical chemotherapeutic agents is the design of new compounds, conceived to inhibit P-gp and to exert other anti-cancer effects, either alone or combined with chemotherapy [10]. The work of Szemerédi et al. follows this direction. Using a drug repurposing approach, i.e., starting from phenothiazines combined with the selenoanhydride and selenoester moieties known for their synergistic effects with substrates of Pgp, the authors synthesized two libraries of ketone-containing and cyano-containing selenoesters. Notably, all compounds displayed more cytotoxic activity against cancer cell lines, either sensitive or drug-resistant, than on non-transformed cell lines, indicating a potentially good therapeutic window. Most compounds are multi-target drugs, because, in addition to inhibiting P-gp, they induce apoptosis and reduce cell migration, and specific keto-and cyanoesters were synergistic with doxorubicin, confirming the effective overcoming of P-gp-mediated resistance [24]. This work is a good example of building new combination treatments against MDR tumors, exploiting a drug repurposing approach.

One of the main causes of failure in the treatment of MDR cancers is specially related to resistance to platinum coordination complexes commonly used in first-line treatments, such as cisplatin or carboplatin. The development of new classes of compounds is therefore necessary to take advantage of multi-targeting mechanisms of action, different from the well-known DNA-binding way of action of platinum agents. From this perspective, bio-organometallic compounds have been widely studied and especially new metal complex architectures based on chemically similar neighboring transition metals, such as ruthenium [25] and iron [26]. Therefore, the article about organoruthenium complexes by Kladnik et al. [25] explores a new family bearing structurally modified pyrithione ligands with extended aromatic scaffolds and their mechanism of action applied to ovarian cancer. The nature of the monodentate site was confirmed to be crucial in the activation and the mechanism of action appeared to be unique from that of cisplatin. The review of Idlas et al. about ferrocifen-loaded nanocapsules [26] provides an overview of in vitro and in vivo studies performed with ferrocifen-loaded lipid nanocapsules on several MDR cancers (glioblastoma, breast cancer and metastatic melanoma). An original mechanism dependent on redox properties and generation of active metabolites that can cause the disruption of cell metabolism has been evidenced with these ferrocifens. In both papers, the cytostatic nature of the complexes involving G1 cell cycle arrest, limited apoptosis and the inhibition of thioredoxin reductase enzyme is discussed in a multi-targeting mechanism strategy to combat drug resistance.

In another approach, for such hydrophobic molecules, formulation strategies need to be considered. Lipid nanocapsules, as described in [26], have demonstrated their ability to successfully encapsulate various hydrophobic therapeutic agents, offering the option of surface modification and making it possible to adapt the pharmacological behavior of the nanocarrier. In the research article of Pautu et al. [27], the lipid nanocapsule surface was modified with novel copolymers of N-vinylpyrrolidone and vinylimidazole to impart stealth properties and improve tumor cell entry under acidic conditions. Indeed, thanks to this new coating, replacing the controversial polyethylene glycol (PEG), such nanoparticles were protected from opsonization by complement activation and presented pH-responsive properties, allowing the increase in drug delivery specificity. These stimuli-responsive nanoparticles, which are able to provide various advantages such as a high active drug-loading capacity, low toxicity, targeted delivery, increased uptake by tumor cells and optimized pharmacokinetic patterns of traditional drugs, are expected to overcome MDR in cancer therapy.

It is believed that 90% of drug candidates that enter clinical trials fail during those trials and drug approval processes [28]. One of the challenges of antitumor drug development is the lack of disease-relevant preclinical models [29], particularly models that recreate the tumor complexity and interactions with the tumor microenvironment. The review written by Barbosa et al. [30] highlights the impact of 3D cell culture models on cancer research and drug screening, discussing their advantages and limitations, together with their compatibility with high-throughput drug/compound screenings. This review also offers insights into the adequacy of available readouts provided by 3D cell culture models. In addition, this work also emphasizes the importance of incorporating key microenvironmental elements when designing 3D cell culture models, to improve the predictive value of drug efficacy and safety.

In summary, this Special Issue brings together reviews and original research papers that contribute to understanding and overcoming drug resistance in cancer.
